# Efficacy and safety of negative pressure wound therapy combined with alginate dressings for wound management: a meta-analysis

**DOI:** 10.3389/fmed.2026.1772888

**Published:** 2026-04-01

**Authors:** Zhenhua He, Hongmei Yang

**Affiliations:** 1 School of Medicine and Health, Shaoxing Institute of Technology, Shaoxing, Zhejiang, China; 2 The Central Hospital of Enshi Tujia and Miao Autonomous Prefecture, Enshi, Hubei, China

**Keywords:** alginate, care, dressing, management, negative pressure wound therapy, nursing, wound

## Abstract

**Background:**

Wound management remains a clinical challenge, with single-modality therapies such as negative pressure wound therapy (NPWT) or conventional dressings often failing to optimize healing efficiency and quality. This study aimed to systematically evaluate the clinical efficacy of NPWT combined with alginate dressings in wound treatment, providing evidence for clinical decision-making.

**Methods:**

A comprehensive search was conducted across Chinese and English databases from database inception to 30 November 2025. Randomized controlled trials (RCTs) comparing NPWT combined with alginate dressings (combined group) versus NPWT alone or routine dressing changes (control group) were included. Two researchers independently performed literature screening, data extraction, and risk of bias assessment using the Cochrane RoB 2.0 tool. Meta-analysis was conducted with RevMan 5.4 software.

**Results:**

A total of 11 RCTs involving 902 participants were included. Pooled results showed that the combined group significantly improved wound healing rate (OR = 2.64, 95% CI 1.60–4.36, I^2^ = 30%) and Grade-A healing rate (OR = 4.69, 95% CI 2.56–8.57, I^2^ = 0%), shortened healing time (days) (MD = −9.00, 95% CI -9.41 to −8.58, I^2^ = 83%), reduced dressing change frequency (MD = −2.54, 95% CI -2.92 to −2.17, I^2^ = 77%), and decreased wound pH value (MD = −0.82, 95% CI -1.08 to −0.57, I^2^ = 61%) compared with the control group. Heterogeneity was moderate to high for partial outcomes, mainly attributed to variations in NPWT parameters (100–500 mmHg) and dressing protocols. No significant publication bias was detected (all *p* > 0.05).

**Conclusion:**

This meta-analysis finds that NPWT combined with alginate dressings significantly improves wound healing outcomes compared to monotherapy, with benefits observed primarily in hospital-based settings. While this combined regimen should be prioritized for complex or chronic wounds, judicious application of parameters—including careful monitoring at higher negative pressures—is warranted, and further health economic evaluation and standardized safety reporting are needed to optimize clinical decision-making.

## Introduction

Wound healing disorders, including chronic wounds such as pressure ulcers, diabetic foot ulcers, and traumatic wounds with delayed healing, have become a major global clinical challenge, imposing a heavy burden on healthcare systems and patients’ quality of life (1). According to clinical epidemiological data, the incidence of chronic wounds has shown a gradual upward trend in recent years, attributed to factors such as aging populations, the increasing prevalence of diabetes and cardiovascular diseases, and improvements in trauma rescue success rates (2–4). These wounds are often characterized by prolonged healing cycles, high risks of infection, and frequent recurrence, which not only increase medical costs but also lead to physical pain, psychological distress, and even disability in severe cases, highlighting an urgent need for effective therapeutic interventions (5, 6).

In clinical practice, wound management strategies have evolved significantly, with negative pressure wound therapy (NPWT) and alginate dressings emerging as two widely used and evidence-based approaches. NPWT, which promotes wound healing by creating a controlled negative pressure environment to remove exudate, reduce edema, and enhance blood perfusion, has been proven superior to traditional dressing changes in accelerating granulation tissue growth and reducing infection rates (7, 8). Alginate dressings, derived from natural algal polysaccharides, exhibit excellent water absorption, biodegradability, and biocompatibility, forming a moist healing environment that supports cell proliferation while minimizing tissue adhesion and pain during dressing changes (9, 10). However, single-modality therapy often faces limitations: NPWT alone may be insufficient to manage excessive exudate in highly exudative wounds, and alginate dressings alone lack the mechanical stimulation and exudate clearance capabilities of negative pressure (11, 12). The theoretical synergy of combining these two modalities—utilizing NPWT’s mechanical effects and alginate dressings’ moist healing advantages—has gained attention, but existing clinical studies on their combined application have shown inconsistent results regarding efficacy and safety (13, 14).

Given the conflicting evidence from individual clinical trials and the lack of a comprehensive synthesis of available data, a systematic evaluation and meta-analysis are urgently needed to clarify the overall efficacy and safety of NPWT combined with alginate dressings in wound treatment. Individual studies are often limited by small sample sizes, single-center designs, and variation in intervention protocols (e.g., negative pressure parameters and dressing change frequency), which may lead to biased or underpowered conclusions. A meta-analysis, by integrating data from multiple high-quality studies, can increase statistical power, reduce random error, and provide a more reliable assessment of the combined therapy’s clinical value. This research is of great practical significance: it can provide evidence-based guidance for clinicians in selecting optimal wound management strategies, improve treatment outcomes for patients with difficult-to-heal wounds, and offer a reference for healthcare decision-making regarding resource allocation and clinical practice guidelines. Therefore, this study aims to conduct a rigorous meta-analysis to evaluate the efficacy and safety of NPWT combined with alginate dressings compared to conventional therapy or single-modality treatment, to provide high-level evidence for clinical practice.

## Methods

### Study design and ethical considerations

This study adopted a systematic review and meta-analysis design, strictly conducted in accordance with the Preferred Reporting Items for Systematic Reviews and Meta-Analyses (PRISMA) statement (15). The study population included patients with various types of wounds who received negative pressure wound therapy (NPWT) combined with alginate dressings, including but not limited to pressure ulcers, diabetic foot ulcers, and traumatic wounds. The control group consisted of patients treated with routine dressing changes, NPWT alone, or alginate dressings alone. As this study was a secondary analysis based on published literature and did not directly involve human subjects, separate ethical approval was not required.

### Search strategy

A comprehensive and unbiased search strategy was developed to ensure the inclusion of all relevant studies. The search covered core Chinese and English databases. Chinese databases included China National Knowledge Infrastructure (CNKI), Wanfang Data Knowledge Service Platform, VIP Chinese Science and Technology Periodical Database (VIP), and Chinese Biomedical Literature Database (CBM). English databases included PubMed, Embase, Cochrane Library, and Web of Science. The search period was limited from the establishment of each database to 30 November 2025, to ensure the inclusion of the latest published relevant studies. Chinese search terms included “negative pressure wound therapy,” “negative pressure drainage,” “alginate dressings,” “wound,” “ulcer,” and “randomized controlled trial.” English search terms included “Negative Pressure Wound Therapy,” “NPWT,” “Alginate Dressings,” “Wound,” “Ulcer,” “Randomized Controlled Trial,” and “RCT.” A combination of subject terms and free words was used for searching, and search strategies were adjusted according to the retrieval rules of different databases to improve search accuracy. Additionally, reference lists of included studies and relevant review articles were manually searched to supplement potentially eligible studies that might have been missed by database searches. For gray literature such as conference abstracts and unpublished studies, complete data were obtained by contacting the corresponding authors to ensure comprehensiveness and unbiasedness of the search.

### Inclusion and exclusion criteria

Eligible studies were published RCTs, regardless of blinding or allocation concealment. Participants were adults aged ≥18 years with acute or chronic wounds, including but not limited to pressure ulcers, diabetic foot ulcers, traumatic wounds, and postoperative wounds with delayed healing (e.g., poor surgical incision healing), with no restrictions on wound duration or baseline area. The experimental group received NPWT combined with alginate dressings, with clear reporting of NPWT parameters (pressure, duration, and frequency) and alginate dressing change protocols. Comparators included routine dressing changes (e.g., gauze and povidone-iodine disinfection), NPWT alone, or alginate dressings alone, with detailed control intervention descriptions. All studies reported at least one extractable efficacy or safety indicator (e.g., wound healing rate, healing time, Grade-A healing rate, dressing change frequency, wound pH, and adverse reaction rate) and were available as full-text articles in Chinese or English.

Excluded studies included non-RCT designs (e.g., cohort studies, case–control studies, case reports, cross-sectional studies, reviews, and commentaries), duplicate/highly overlapping publications (only the latest or most data-complete version retained), and studies with inadequate design or unrecoverable missing key data (e.g., sample size and outcome values) after author contact. Also excluded were basic research (e.g., animal/*in vitro* cell studies), studies involving minors (aged years) or pregnant/lactating women without separate subgroup data, and studies with unclear NPWT-alginate combination use, no substantial experimental-control intervention differences, inaccessible full text, irregular data formats, obvious falsification, or logical contradictions.

### Literature screening

Two researchers independently conducted literature screening in accordance with the aforementioned inclusion and exclusion criteria, following a two-stage process: initial screening and full-text screening. During initial screening, literature that was clearly inconsistent with the criteria was rapidly excluded by reading titles and abstracts. For literature that passed the initial screening, full texts were reviewed in detail during the full-text screening stage to verify compliance with all inclusion criteria, and the final set of studies included in the analysis was determined. In case of discrepancies between the two researchers, consensus was first reached through face-to-face discussion. If no agreement could be reached, a third senior researcher was consulted for arbitration. EndNote X9 software was used to manage retrieved literature and automatically remove duplicate studies, ensuring the efficiency and accuracy of the screening process.

### Quality assessment

The Cochrane Collaboration’s Risk of Bias Assessment Tool (RoB 2.0) (16) was used for comprehensive quality evaluation of the included RCTs. The evaluation covered seven core domains: random sequence generation, allocation concealment, blinding of participants and personnel, blinding of outcome assessment, completeness of outcome data, selective reporting of results, and other potential biases. Each domain was classified into three levels: “low risk of bias,” “high risk of bias,” and “some concerns.” The classification was based on the descriptions of relevant study design and implementation details in the original studies. Quality assessment was independently completed by two researchers, who received unified training prior to the assessment to ensure consistency in evaluation standards. After the assessment, the results were cross-checked. Any discrepancies were resolved by reviewing the original literature, verifying evaluation criteria, and conducting discussions, ultimately forming consistent quality assessment results to provide a reliable basis for subsequent heterogeneity analysis and result interpretation.

### Data collection

A standardized form was used for independent data extraction by two researchers, covering basic study information (first author, publication year, country/region, sample size, and research institution), participant baseline characteristics (age, gender distribution, wound type/composition, wound duration, and baseline wound area), intervention details, outcome data, and risk of bias information (randomization method, allocation concealment, and blinding details). Missing or unclear data were supplemented by full-text review or email contact with corresponding authors. Unrecoverable data led to decisions on inclusion or descriptive analysis based on data importance. Extracted data were cross-checked via double-entry to ensure accuracy, with inconsistencies resolved by verifying original literature.

### Statistical analysis

Statistical analysis was performed using RevMan 5.4. Heterogeneity among studies was assessed via χ^2^ test and I^2^ statistic for dichotomous outcomes (e.g., wound healing rate, adverse reaction rate, and Grade-A healing rate) with *α* = 0.1. For outcomes with low heterogeneity (*p* > 0.1 and I^2^ < 50%), a fixed-effects model was applied for meta-analysis. In cases of moderate-to-high heterogeneity (*p* ≤ 0.1 or I^2^ ≥ 50%), we first explored potential sources through subgroup analyses (predefined by wound type, sample size, intervention duration, and NPWT parameters) and sensitivity analyses (sequentially excluding individual studies). Meta-regression was considered to examine the influence of NPWT pressure on outcomes such as healing time and dressing change frequency; however, it was not performed due to the limited number of studies available for these outcomes (k = 10 and k = 8, respectively) and incomplete reporting of pressure parameters across included studies. If heterogeneity remained unexplained but no significant clinical inconsistency was observed, a random-effects model was adopted. Dichotomous outcomes were reported as odds ratios (OR) with 95% confidence intervals [95% CI]; continuous outcomes (e.g., healing time, dressing change frequency, and wound pH) were reported as mean difference (MD) with 95% CI or standardized mean difference (SMD) with 95% CI. Publication bias was evaluated via funnel plots and Egger’s test (P 05 indicating potential bias), corrected by the trim-and-fill method if needed. All tests were two-tailed with *α* = 0.05.

## Results

### Study selection

**Figure 1:**
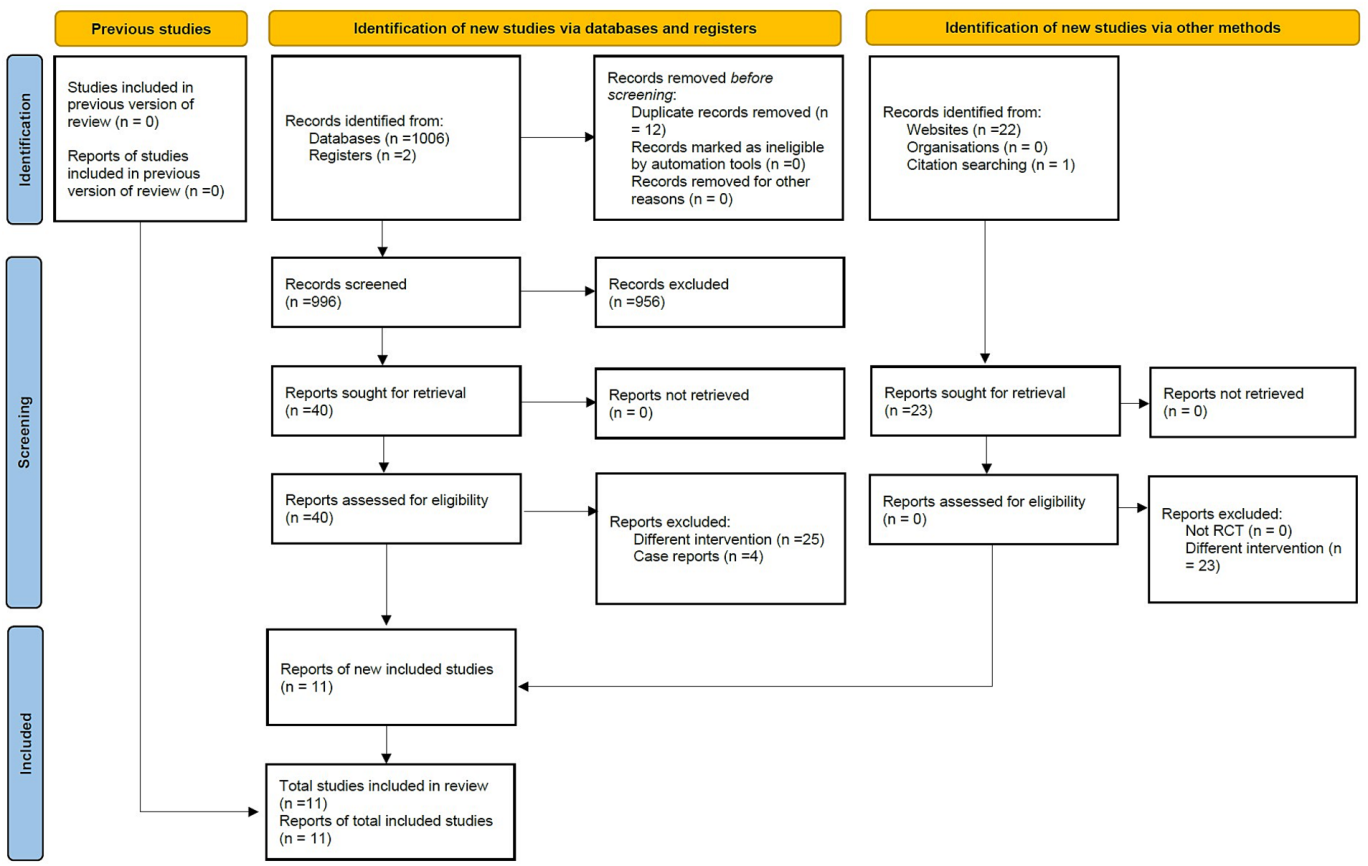
The PRISMA flow diagram of study selection.

A total of 1,008 records were retrieved through database searches, with an additional 23 records supplemented via other sources. After removing 12 duplicate records, 996 records underwent title/abstract screening, and 956 records were excluded. Full texts of 40 studies were obtained and evaluated, among which 25 were excluded due to inconsistent intervention measures and 4 due to being case reports. Finally, 11 RCTs (17–27) were included in this meta-analysis (Figure 1).

### Basic characteristics of included studies

A total of 11 RCTs (17–27), published between 2013 and 2025, were included, with all studies conducted in China (Table 1). The total number of participants across all studies was 902, with 451 in the combined group (NPWT plus alginate dressings) and 451 in the control group. Control interventions mainly consisted of NPWT alone or routine wound dressing changes. Negative pressure intensity varied across studies, ranging from approximately 100 to 500 mmHg. Based on the intervention protocols described (e.g., multiple daily sessions and requirements for close wound monitoring), NPWT was inferred to be primarily administered in hospital settings, although the original studies did not explicitly specify the care environment. The most common intervention frequency and course were “30 minutes per session, 3–4 times daily for 7 consecutive days,” while some studies adopted protocols based on daily cumulative duration or adjustments according to wound conditions. Notably, for the outcome of Grade-A wound healing reported in four studies (19, 21, 25, 27), the definition was consistent across all trials, applying standard clinical criteria of wound healing with good approximation and absence of redness, swelling, exudation, or infection.

**Table 1:** The characteristics of included RCTs.

RCT	Sample size		Age		Intervention		Negative pressure	Frequency/duration
	Combined group	Control group	Combined group	Control group	Combined group	Control group		
Chen 2020 (17)	44	44	52.4 ± 4.1	46.7 ± 3.5	NPWT plus alginate dressings	NPWT	350~400	30 min per session, 3 times daily, for 7 consecutive days
Du 2020 (18)	30	30	46.6 ± 9.9	47.5 ± 9.4	NPWT plus alginate dressings	NPWT	300~450	30 min per session, 4 times daily, for 7 consecutive days
He 2019 (19)	32	28	39.21 ± 7.19	40.51 ± 8.29	NPWT plus alginate dressings	NPWT	125~450	30 min per session, for 7 consecutive days
Hu 2018 (20)	45	45	47.2 ± 2.6	46.5 ± 2.5	NPWT plus alginate dressings	Routine wound dressing changes	100	Administered once every 3 consecutive days
Hu 2020 (21)	40	40	34.97 ± 6.53	33.65 ± 7.43	NPWT plus alginate dressings	Routine wound dressing changes	350	30 min per session, 4 times daily
Jia 2021 (22)	29	29	45.99 ± 7.12	45.65 ± 7.64	NPWT plus alginate dressings	Routine wound dressing changes	125~450	During negative pressure suction, closely monitor the patient’s wound status; if any abnormalities occur, intervene promptly. After granulation tissue proliferation, suspend negative pressure suction and continue dressing changes with alginate and foam dressings until wound healing is achieved.
Li 2013 (23)	33	33	12 ~ 72	12 ~ 72	NPWT plus alginate dressings	Routine wound dressing changes	300~450	30 min per session, 4 times daily, for 7 consecutive days
Luo 2025 (24)	46	46	34.2 ± 10.0	33.8 ± 9.4	NPWT plus alginate dressings	Routine wound dressing changes	125~450	6 h/d, 3 h each in the morning and afternoon
Qi 2016 (25)	30	30	18 ~ 70	17 ~ 68	NPWT plus alginate dressings	Routine wound dressing changes	300~450	30 min per session, 4 times daily, for 7 consecutive days
Shang 2024 (26)	36	36	66.82 ± 3.43	66.23 ± 3.61	NPWT plus alginate dressings	Routine wound dressing changes	450~500	30 min per session, 3 times daily, for 7 days
Yang 2016 (27)	50	50	40.5 ± 8.3	39.2 ± 7.2	NPWT plus alginate dressings	NPWT	125~450	30 min per session, for 7 consecutive days

NPWT, negative pressure wound therapy; RCT, randomized controlled trials.

### Risk of bias assessment

**Figure 2:**
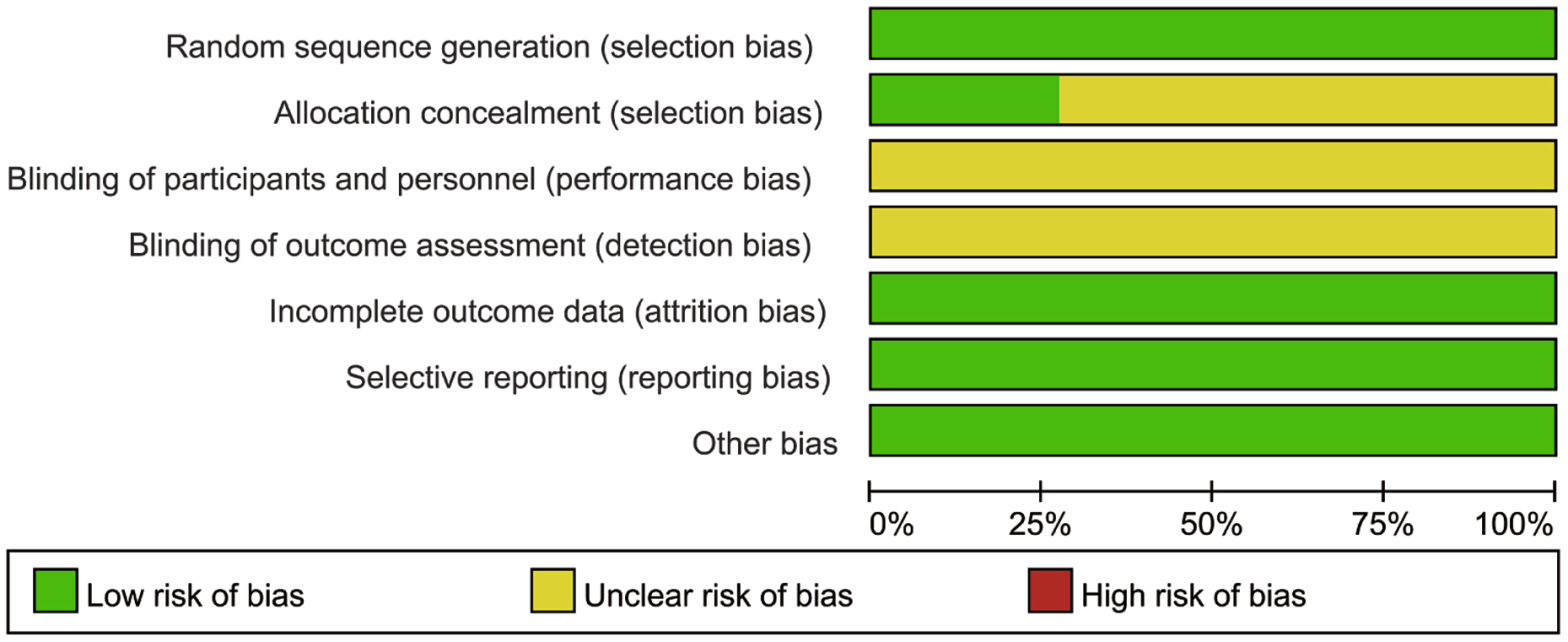
Risk of bias graph.

**Figure 3:**
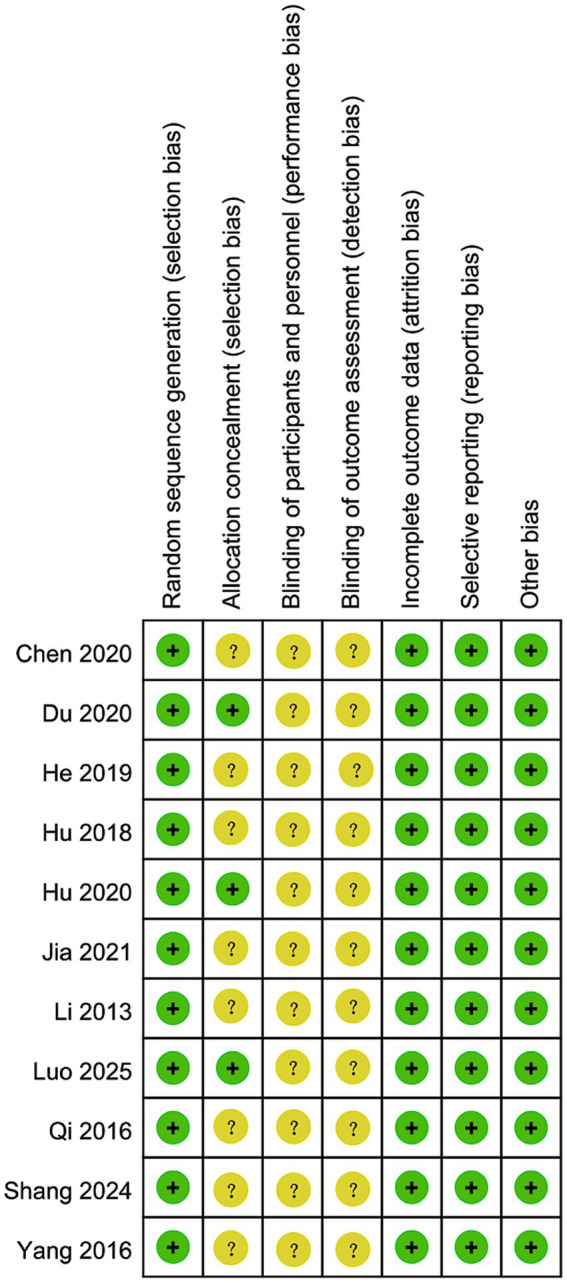
Risk of bias summary.

Overall, all studies (17–27) were rated as low risk for random sequence generation. Most studies were classified as “unclear” for allocation concealment, with only a few reporting adequate methods. Blinding of participants and personnel, as well as blinding of outcome assessment, were predominantly rated as “unclear” across studies. Attrition/incomplete outcome data, selective reporting, and other biases were mostly rated as low risk (Figure 2). The dimension-specific risk of bias assessment results for individual studies were consistent with the above findings (Figure 3).

### Meta-analysis results

**Figure 4:**
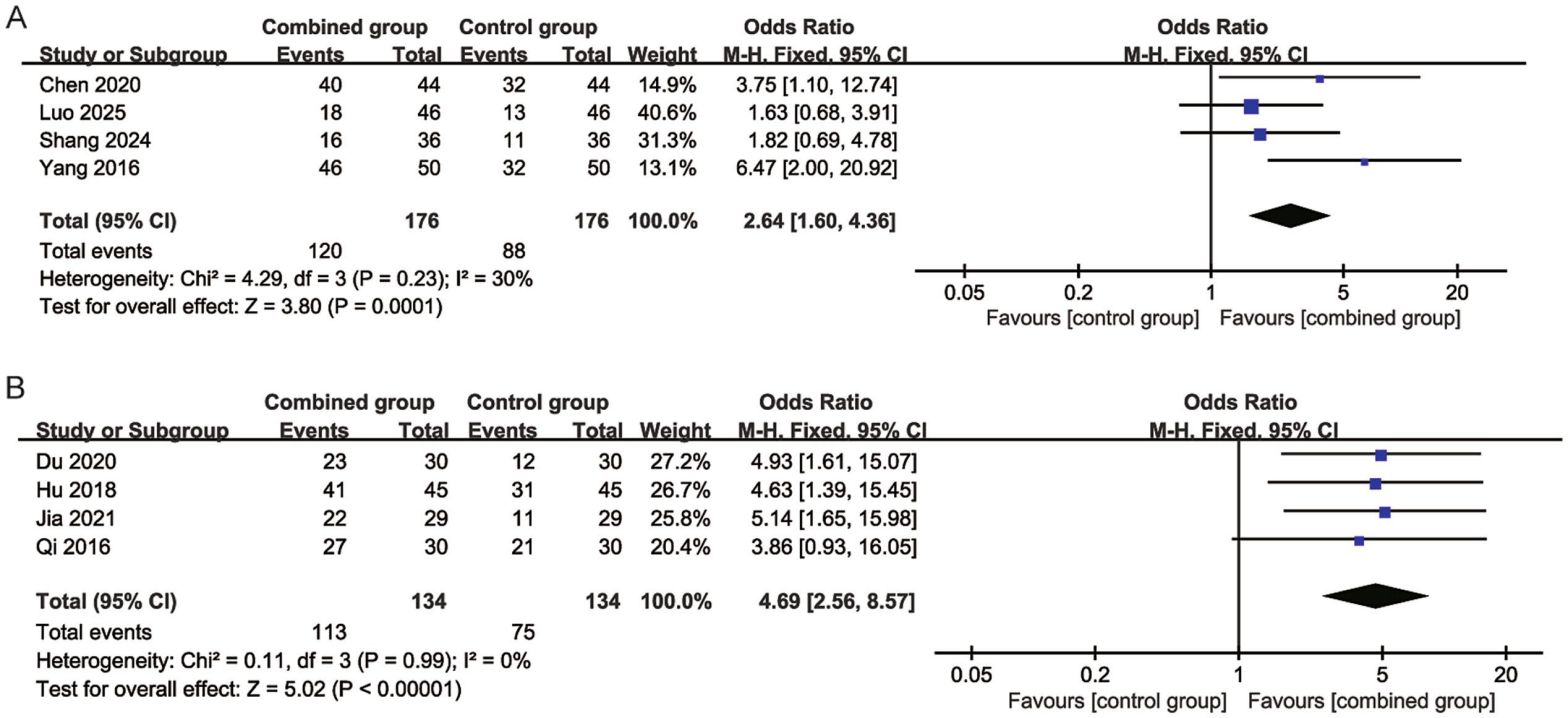
Forest plots for wound healing rate and Grade-A wound healing status. **(A)** Forest plot for wound healing rate. **(B)** Forest plot for grade A wound healing status.

*Wound healing rate*: Four studies involving 176 participants in the combined group and 176 in the control group were included. Pooled analysis using a fixed-effects model showed that NPWT combined with alginate dressings significantly improved the wound healing rate (OR = 2.64, 95% CI 1.60–4.36) with moderate heterogeneity (I^2^ = 30%) (Figure 4A).

*Grade-A wound healing status*: Four studies with 134 participants in each group were included. The pooled effect indicated that the combined group was superior to the control group in achieving Grade-A wound healing (OR = 4.69, 95% CI 2.56–8.57) with low heterogeneity (I^2^ = 0%) (Figure 4B).

**Figure 5:**
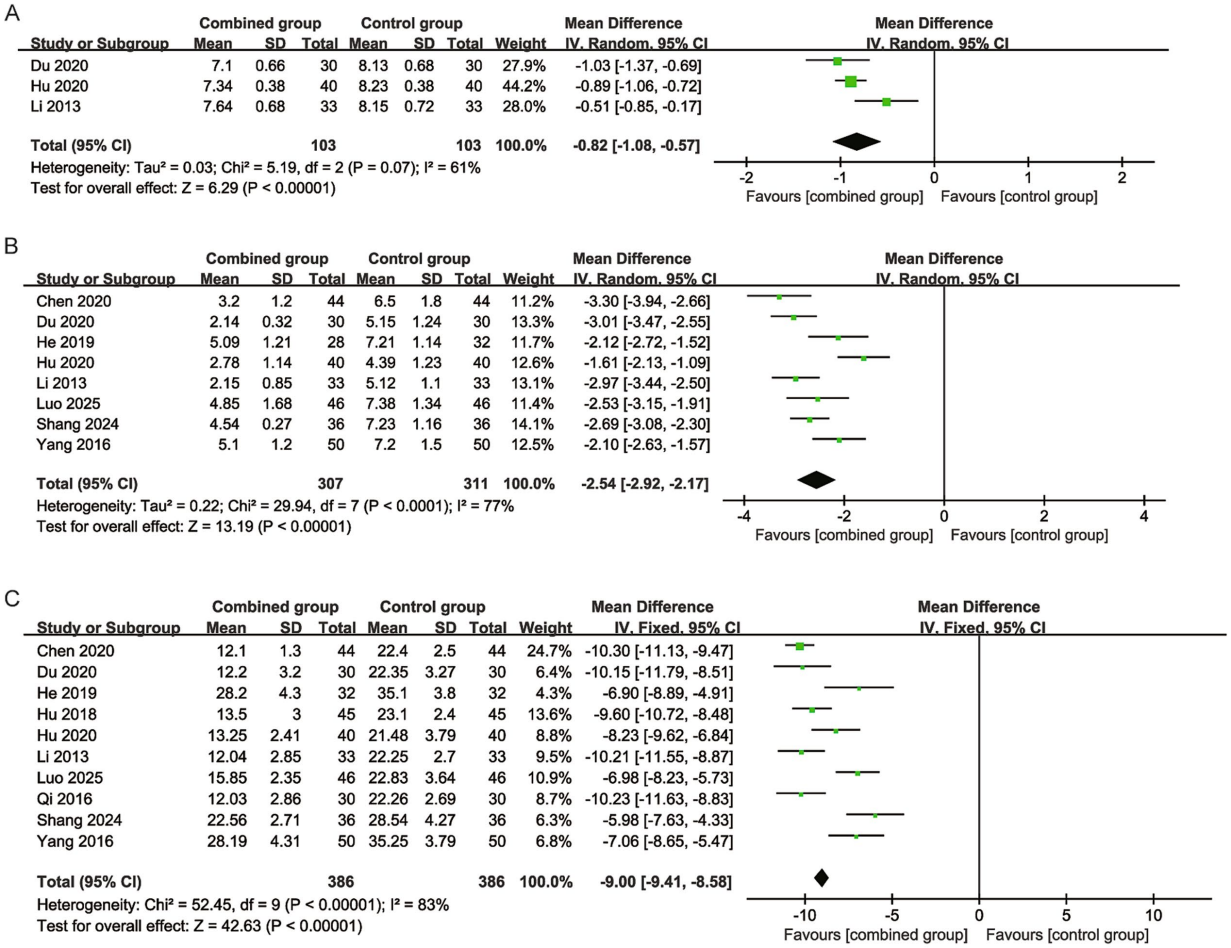
Forest plots for wound pH value, frequency of wound dressing changes, and wound healing time (days). **(A)** Forest plot for wound pH value. **(B)** Forest plot for frequency of wound dressing changes. **(C)** Forest plot for wound healing time.

*Wound pH value*: Three studies involving 103 participants in each group were included. Pooled analysis using a random-effects model revealed that the combined group had a significantly lower wound pH value (MD = −0.82, 95% CI -1.08 to −0.57) with moderate heterogeneity (I^2^ = 61%) (Figure 5A).

*Frequency of dressing changes*: Eight studies with 307 participants in the combined group and 311 in the control group were included. The random-effects model showed that the combined group required significantly fewer dressing changes (MD = −2.54, 95% CI -2.92 to −2.17) with high heterogeneity (I^2^ = 77%) (Figure 5B).

*Wound healing time*: In total, 10 studies involving 386 participants in each group were included. Pooled results demonstrated that the combined group had a significantly shorter wound healing time (days) (MD = −9.00, 95% CI −9.41 to −8.58) with high heterogeneity (I^2^ = 83%) (Figure 5C).

### Publication bias

**Figure 6:**
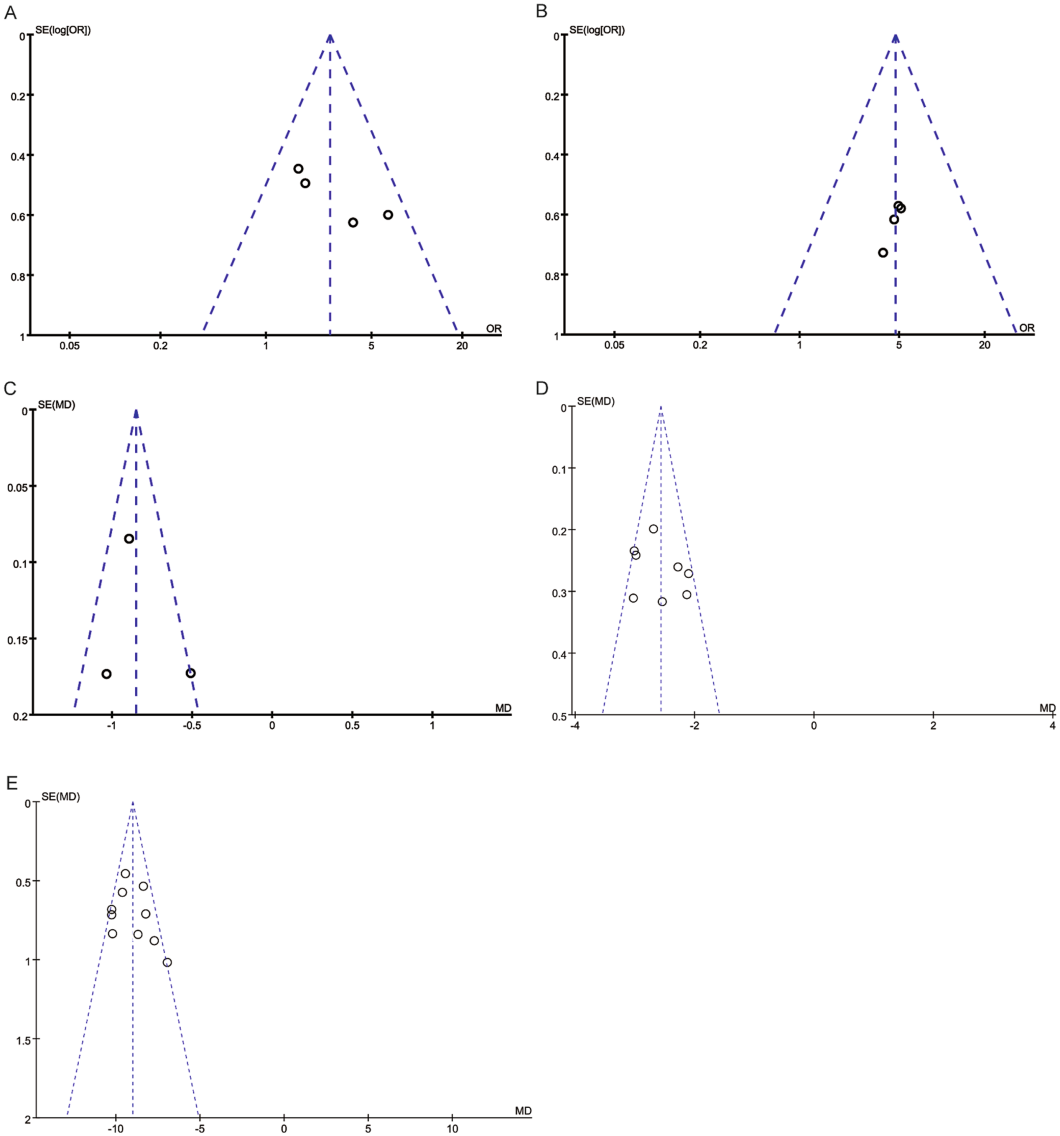
Funnel plots for analyzed outcomes. **(A)** Funnel plot for wound healing rate. **(B)** Funnel plot for grade A wound healing status. **(C)** Funnel plot for wound pH value. **(D)** Funnel plot for frequency of wound dressing changes. **(E)** Funnel plot for wound healing time.

The number of studies included in the funnel plots was generally small, with slight deviations in the distribution of some outcome points (Figure 6). Egger regression analysis indicated no significant publication bias across all meta-analysis results (all *p* > 0.05).

## Discussion

Based on the pooled results of 11 RCTs, this meta-analysis demonstrates that NPWT combined with alginate dressings exhibits significant advantages in improving wound healing-related indicators. Compared with NPWT alone or routine wound dressing changes, the combined therapy significantly increases the wound healing rate (OR = 2.64) and Grade-A wound healing rate (OR = 4.69), while shortening the wound healing time (days) (MD = −9.00), reducing the frequency of dressing changes (MD = −2.54), and decreasing the wound pH value (MD = −0.82). These results collectively confirm the clinical value of the combined regimen from both efficacy and safety perspectives: the improved healing rate directly reduces the risk of complications such as wound infection and ulcer recurrence, while the reduced frequency of dressing changes not only alleviates the pain and economic burden on patients but also reduces the workload of medical staff. Notably, the significant improvement in Grade-A wound healing rate indicates enhanced wound healing quality, which helps reduce long-term adverse outcomes such as scar hyperplasia and skin dysfunction, and is of great significance for improving patients’ quality of life. It should be noted that, based on the intervention protocols described in the included studies (e.g., multiple daily sessions and requirements for close wound monitoring), NPWT was primarily administered in hospital settings. Consequently, while the findings demonstrate clear efficacy, their direct extrapolation to ambulatory or home-care settings should be approached with caution, and further research is warranted to evaluate the feasibility and effectiveness of the combined therapy in such environments.

The synergistic effect between NPWT and alginate dressings is the core reason for its superior efficacy over single-modality therapy. NPWT creates a favorable mechanical environment for wound healing by removing wound exudate, reducing edema, promoting local blood circulation, and enhancing granulation tissue proliferation through a controlled negative pressure environment (28–30). As a natural biomaterial, alginate dressings possess high water absorption and biocompatibility, capable of absorbing a large amount of exudate to form a gel-like protective membrane, maintaining a moist wound environment, and reducing adhesion between the dressing and the wound (31). Meanwhile, their degradation products can regulate the wound pH value—this study shows that the wound pH value in the combined group was significantly lower (MD = −0.82), and an acidic environment helps inhibit bacterial proliferation, promote fibroblast activity, and epithelial cell migration, further accelerating the healing process (32). In addition, the mechanical drainage effect of NPWT can avoid the frequent replacement of alginate dressings caused by excessive absorption of exudate, while alginate dressings can compensate for the deficiencies of NPWT in local wound moisturization and antibacterial activity (33, 34). The two form a functional complementarity, jointly optimizing the pathophysiological microenvironment for wound healing.

The results of this study are consistent with the conclusions of multiple previous clinical studies. For example, RCTs by Chen et al. (17) and Du et al. (18) both found that NPWT combined with alginate dressings shortened the healing time by more than 30% compared with NPWT alone in patients with traumatic wounds and poor surgical incision healing, which is highly consistent with the pooled result of MD = −9.00 for healing time in this study. However, some studies have reported inconsistencies: certain studies did not observe a significant effect of combined therapy on pH regulation, which may be related to the heterogeneity of NPWT negative pressure parameters (100–500 mmHg) and dressing change protocols among the included studies. It is worth noting that the upper limit of 500 mmHg, as applied in one included study Shang et al. (26), exceeds the conventional NPWT range (−125 to −150 mmHg). However, this higher pressure was administered under controlled conditions (30-min sessions, three times daily) and was likely tailored to specific wound characteristics (e.g., heavily exudative wounds) where the benefits of rapid exudate removal may outweigh potential risks. Nevertheless, as highlighted in the response to peer review, such higher pressures warrant cautious application with close monitoring for pain, bleeding, or tissue ischemia, and parameter selection should always be individualized based on wound type, tissue tolerance, and patient comfort. The I^2^ values for healing time and dressing change frequency in this study were as high as 83 and 77%, respectively, indicating that differences in intervention details across studies may affect the consistency of results. Furthermore, this study expands the scope of outcome indicators to include key indicators such as Grade-A wound healing rate and wound pH value, more comprehensively revealing the advantages of combined therapy and providing richer evidence to support clinical decision-making.

Based on the results of this study and combined with clinical practice needs, the following optimization suggestions are proposed. In terms of treatment strategy, for wounds requiring long-term management, such as traumatic wounds, poor surgical incision healing, and pressure ulcers, the combination therapy of NPWT and alginate dressings should be prioritized (35). The recommended conventional intervention parameters are 30 min per session, 3–4 times per day for 7 consecutive days, and the negative pressure intensity can be adjusted to 125–450 mmHg according to the wound type (this range is the mainstream choice in the included studies, with more reference value for efficacy and safety). For wounds with excessive exudate, the frequency of alginate dressing replacement can be moderately increased, and the efficient drainage function of NPWT can be utilized to further improve treatment outcomes (36). At the level of nursing measures, close monitoring of wound status is required during treatment, and negative pressure parameters and dressing types should be dynamically adjusted according to the progress of granulation tissue proliferation (37). Gentle operation techniques should be adopted during dressing changes to avoid damaging newly formed granulation tissue, and changes in wound pH value should be recorded regularly to provide objective evidence for evaluating healing progress. In the construction of the management system, it is necessary to establish standardized operating procedures for combined therapy and conduct special training for medical staff on key links such as NPWT parameter setting and dressing change skills. Patient education should be strengthened, focusing on guiding patients on posture management, wound protection points, and abnormal situation identification methods during home care to improve treatment compliance (38). Medical resources should be reasonably allocated based on health economic evidence, and the combined therapy should be prioritized for high-risk populations such as patients with diabetic foot ulcers and pressure ulcers to achieve optimal control of medical costs (39).

### Limitations and future directions

This study has several limitations that should be considered when interpreting the findings. First, regarding methodological transparency, although the review protocol was finalized prior to data extraction, it was not prospectively registered in a public database (e.g., PROSPERO), which represents a limitation in terms of research transparency and reproducibility. Second, only 11 RCTs were included, all of which were conducted in China, with individual study sample sizes ranging from 58 to 100 cases. The relatively small overall sample size and single-country setting may introduce selection bias, and caution should be exercised when extrapolating these findings to other countries, regions, or healthcare systems with different wound care practices. Third, the heterogeneity of intervention measures was substantial, with considerable variation across studies in key parameters such as NPWT negative pressure intensity (ranging from 100 to 500 mmHg), session duration and frequency, and alginate dressing models. Although subgroup and sensitivity analyses were performed to explore potential sources of heterogeneity, the high I^2^ values for outcomes such as healing time (83%) and dressing change frequency (77%) remained partially unexplained, likely attributable to the limited number of studies and incomplete reporting of pressure parameters that precluded meta-regression. Fourth, the reporting on safety outcomes was notably insufficient. Upon re-examination of the included studies, explicit and extractable data on adverse events—such as bleeding, infection, pain, or tissue damage—were largely absent. Most studies provided only qualitative statements (e.g., “no significant complications observed”), and none reported safety data stratified by pressure levels. Consequently, a pooled analysis of adverse events was not feasible, and the safety profile of the combined therapy, particularly at higher negative pressures (e.g., 450–500 mmHg), remains to be rigorously established. Fifth, the follow-up periods for outcome assessment lacked uniformity, and several studies did not report long-term healing quality indicators (e.g., scar formation and wound recurrence rate), limiting our ability to evaluate the sustained benefits of combined therapy. Sixth, as the reviewers rightly noted, a formal health economic evaluation was beyond the scope of this study because the included RCTs did not report detailed cost data. While our findings of significantly shortened healing time (MD = −9.00 days) and reduced dressing change frequency (MD = −2.54) serve as clinical proxies suggesting potential cost savings, direct evidence on the cost-effectiveness of NPWT combined with alginate dressings compared to monotherapy remains lacking. Finally, although Egger’s test did not indicate significant publication bias, visual inspection of funnel plots revealed some asymmetry, which may be related to unpublished studies with negative results.

Based on these limitations, future research should prioritize the following: (1) prospective registration of systematic review protocols to enhance methodological transparency; (2) multi-center, large-sample RCTs with standardized intervention protocols (including uniform NPWT parameters and dressing regimens) to reduce heterogeneity and improve generalizability; (3) systematic and transparent reporting of adverse events using standardized classification systems, enabling comprehensive risk–benefit assessment across different pressure levels; (4) extended follow-up periods incorporating long-term outcomes such as scar quality, recurrence rates, and patient-reported quality of life; (5) formal health economic evaluations to validate the hypothesized cost savings suggested by clinical efficacy outcomes and to inform rational allocation of healthcare resources; and (6) subgroup analyses in special populations (e.g., diabetic patients, elderly individuals, and those with infected wounds) to refine patient selection and optimize treatment protocols.

## Conclusion

This meta-analysis provides evidence that the combination of NPWT and alginate dressings significantly enhances wound healing outcomes compared to monotherapy (NPWT alone or conventional dressings). The combined regimen not only improves overall and Grade-A healing rates but also shortens healing time, reduces dressing change frequency, and optimizes the wound microenvironment by lowering pH. These clinical benefits translate into reduced patient discomfort, lower treatment burden, and potentially favorable economic implications, although formal cost-effectiveness analyses are warranted. Given that the included studies primarily administered NPWT in hospital settings, the generalizability of these findings to ambulatory or home-care environments requires further investigation. Moreover, while higher negative pressures (up to 500 mmHg) were employed in select cases—likely justified by specific wound characteristics such as heavy exudate—such settings should be applied with caution, with close monitoring for adverse events and individualized titration based on tissue tolerance and patient comfort. In clinical practice, this combined therapy should be prioritized for complex or chronic wounds (e.g., traumatic wounds, pressure ulcers, diabetic foot ulcers, and postoperative wounds with delayed healing) using standardized parameters (e.g., 125–450 mmHg negative pressure, 30-min sessions 3–4 times daily for 7 consecutive days) and tailored adjustments according to wound exudate, granulation tissue formation, and patient response. Future research should include prospective protocol registration, multicenter large-sample RCTs with unified intervention protocols, systematic safety reporting across pressure levels, long-term follow-up assessing recurrence and quality of life, and rigorous health economic evaluations to inform evidence-based resource allocation.

## Data Availability

The original contributions presented in the study are included in the article/supplementary material, further inquiries can be directed to the corresponding author.
